# Predictors of Acute Kidney Injury in Geriatric Patients Undergoing Total Knee Replacement Surgery

**DOI:** 10.5812/ijem.16713

**Published:** 2014-07-01

**Authors:** Vishal Sehgal, Sukhminder Jit Singh Bajwa, Rinku sehgal, Jeremiah Eagan, Praveen Reddy, Samuel M. Lesko

**Affiliations:** 1 Department of Internal Medicine, University of South Carolina.Greenville, South Carolina, USA; 2 Department of Anesthesiology and Intensive Care Medicine, Gian Sagar Medical College, Banur, Patiala, Punjab, India; 3 Department of Internal Medicine, Wright Center, Scranton, USA; 4 Department of Nephrology, Regional Hospital, Scranton, USA; 5 Northeast Regional Cancer Institute, University of Scranton, Scranton, USA

**Keywords:** Acute Kidney Injury, Geriatrics, Chronic Kidney Disease, Diabetes Mellitus, Total Knee Replacement, Health Care Reform

## Abstract

**Background:**

Few studies have focused on patients’ characteristics that affects acute kidney injury (AKI) after total knee replacement surgery (TKR).

**Objectives:**

The primary objective of this retrospective study was to identify patients’ characteristics associated with AKI after TKR.

**Patients and Methods:**

Between January 2008 and December 2009, 659 patients with a mean age of 67.1 years (range, 39-99) underwent TKA at Regional Hospital Knee and Hip Institute. Retrospective chart review was done to identify patients’ characteristics that were associated with AKI after TKR. Logistic regression was used to evaluate AKI.

**Results:**

AKI occurred in 21.9% of patients. AKI risk decreased between 2008 and 2009 (odds ratio, 0.55; 95% CI, -0.37 to 0.82) but increased with age (P < 0.001), diabetes mellitus (DM), and angiotensin converting enzyme inhibitors (ACEI)/angiotensin receptor blockers (ARB) use (OR, -1.6; 95% CI, -1.0 to 2.5; and OR, -1.5, 95% CI, -1.0 to 2.3, respectively). However, the effects of DM and ACEI/ARB use were not independent; when both were included in the regression model, neither was statistically significant, and both ORs were smaller than combined OR.

**Conclusions:**

When examined separately, both DM and preoperative ACEI/ARB use increased the risk of AKI; however these factors were correlated and were not independent predictors of significantly increased risk. Patients with DM have higher tendency to develop AKI and hence, preoperative renal risk stratification should be done in all patients with DM.

## 1. Background

In the light of ever increasing geriatric population, healthcare budgets are constrained. Medicare is expected to cut down healthcare reimbursements in the years to come. The healthcare services would become more and more competitive. This is in sharp contrast to earlier trends when hospitals were reimbursed irrespective of the quality of the services offered. In addition, the definition of quality needs to be redefined as it is not only increased utilization of healthcare resources but also improved outcomes at lower costs. Increasingly, we see aging of the population in the 21^st^ century with the fastest growing population being people over 85 years of age. With the baby boomers moving into geriatric age group, this section of population is going to get even larger. With the aging of the population, more patients with chronic kidney disease (CKD) undergo elective surgeries. Perioperative risk stratification focuses on optimizing the cardiopulmonary function. There is little or no emphasis on renal risk stratification and renal protection strategies (1, 2). With aging, renal function declines significantly. Peak body mass with optimum body physiology is achieved by the age of 30 years; thereafter, there is a gradual decline in it. As the person moves from 30 to 80 years of age, usually 30% of the kidney function is lost (3). The predominant loss is in the renal cortex that affects the most physiologically important nephrons. It results in about 40% lower renal plasma flow in the elderly as compared to the younger adults even when there is no risk factor for kidney disease. A deeper study of the renal diseases based on molecular genetics can help to develop a better knowledge and understanding of these facts (4, 5). To compensate for it, the elderly’s kidneys are maintained in a state of chronic vasodilatation. In perioperative settings, it contributes to increase risk of renal injury in the elderly population especially the septuagenarians and octogenarians. Moreover, when renal injury does occur, the elderly are less likely to recover, especially after an ischemic insult to the kidneys (6-8).

Acute kidney injury (AKI) has traditionally been defined as an abrupt loss of kidney function with resultant accumulation of nitrogenous waste and dysregulation of electrolytes and blood volume. The loss of kidney function may most easily be quantified by measuring the serum creatinine (Cr). It has its own pitfalls as in the elderly who usually have decreased muscle mass, the rise of serum Cr is not proportionate to the loss of kidney function. Cr production decreases with aging; in addition, there is decrease in tubular secretion of Cr. Hence, despite significant decrease in glomerular filtration rate (GFR) in the elderly, Cr may remain within normal range (9). The prevalence of CKD is increased by increased incidence of AKI (10). Moreover, when patients with underlying CKD are exposed to potentially nephrotoxic medications or physiological conditions, they are at increased risk of AKI in the postoperative period. The incidence of such injuries is increased in high-risk patients admitted to the ICUs with comorbidities. This is true even for stage I CKD, which is defined by proteinuria with normal GFR (11-14). In the elderly, AKI is an independent risk factor for hospital mortality and is associated with longer ICU stay, increased morbidity, utilization of resources, and higher mortality at six-month follow-up (15-20). In addition, it is an important prognostic marker for complications during hospitalization in the elderly (21).

Total hip and total knee replacement surgeries (THR and TKR, respectively) for osteoarthritis (OA) are amongst the most successful orthopedic interventions with a positive impact on health-related quality of life. There will be a considerable increase in the number of joint replacement surgeries, even in the low income countries, in the years to come. Physicians will be frequently involved in the medical consultations with patients in the postoperative period of THR/TKR. Current data suggest that postoperative AKI is associated with increased hospital length of stay (22). It leads to loss of revenue to the hospital and decreased patient satisfaction after the elective surgery. We have been seeing substantial AKI in this subset of population. Most of the data on perioperative AKI are from patients with cardiac surgeries (23).

## 2. Objectives

The present study aimed to identify the patients’ characteristics associated with AKI. Diabetes status and angiotensin converting enzyme inhibitors (ACEI) as well as angiotensin receptor blocker (ARB) usage among the elderly patients were of particular interest.

## 3. Patients and Methods

The present study was conducted at a specialty surgical service of a community-based acute care hospital. All patients who underwent TKR surgeries between January 2008 and December 2009, were included in the study regardless of their age, sex, race, comorbidities, and socioeconomic status. AKI was defined as an abrupt (within 48 hours) absolute increase in the serum Cr concentration of ≥ 0.3 mg/dL from baseline. This was based on Acute Kidney Injury Network (AKIN) criteria for AKI (24). Using AKIN, AKI was further subdivided into stages I through III: Stage I, ≥ 0.3mg/dL increase in serum; Stage II, two to 2.9 times the baseline increase in the serum Cr; and stage III, three times the baseline increase in the serum Cr. Urine output was not measured. Included secondary characteristics were age, sex, and marital status. The design of the study was presented to the Institutional Review Board (IRB). The contents of the study included the patients’ characteristics and associated comorbidities. After going through the design of the study, the IRB waived the requirement for informed consent as this was a retrospective review of medical records. Demographic and laboratory data were retrieved from an electronic hospital database. After merging data from the different sources, automated and manual data verification was performed.

### 3.1. Statistical Analysis

All events were recorded from the day of surgery up to the discharge from the acute care hospital. Continuous variables were compared using the independent samples t test and categorical data were compared with the chi square (χ^2^) test. Logistic regression was used to evaluate the association between patients’ characteristics and AKI while controlling for confounding variables. The logistic model included terms for age, sex, year of surgery, DM, and ACEI use. In all tests, P value < 0.05 was considered as statistically significant finding.

## 4. Results

**Figure 1.**
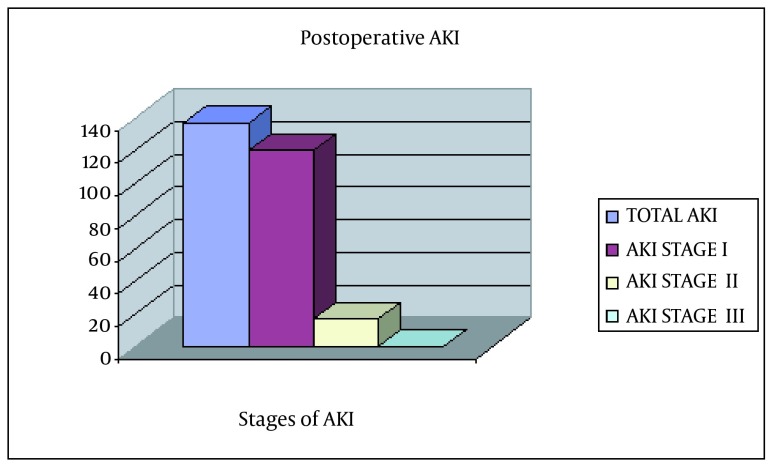
Incidence of Acute Kidney Injury in the Study Acute kidney injury (AKI) was seen in 138 patients. Of these, 120 and 18 had AKI stages I and II, respectively. There was no patient with AKI stage III.

During the study period, a total of 659 (442 females) patients underwent TKR. Of these, 32 were excluded because of missing or incomplete data precluded assessment of renal status. The analysis was limited to 627 patients with sufficient data to evaluate AKI. The characteristics of these patients are shown in Table 1. Overall, 137 (21.9%) patients experienced an episode of AKI (Figure 1) following surgery (stage I, 120; stage II, 17; and no stage III). Patients with AKI were older (mean age, 71.1 ± 10.2 years) than those without AKI (mean age, 66.1 ± 10.2 years; P < 0.001). Risk of AKI did not differ by sex; however, risk of AKI was greater in 2008 than in 2009 (P = 0.002). In addition, risk of AKI was greater among patients with DM and those exposed to ACEIs. Risk of AKI increased by 5% per year of life and, compared to 2008, was approximately 45% lower in 2009. In the bivariate analysis, the risk of AKI was significantly elevated among patients with both DM (OR, 1.58; 95% CI, 1.04-2.40) and ACEI use (OR, 1.55; 95% CI, 1.05-2.30); the risk was greatest among patients with DM who used ACEI (OR, 2.03; 95% CI: 1.20- 3.45). In the multivariable model, mutually exclusive terms were used to indicate patients with DM only (no ACEI use), ACEI use only (without DM), and both DM and ACEI use. In this model, in comparison to patients with neither DM nor ACEI use, the risk of AKI was not significantly elevated in patients with DM alone or those with only ACEI use; however, the risk was nearly two-fold elevated among patients with DM who had also used ACEI (OR, 1.91; 95% CI, 1.10-3.32). The distribution of patients according to the stage of AKI and mutually exclusive categories of DM and ACEI use is shown in Table 2. Risk of AKI was greatest among the 85 patients with DM who used preoperative ACEI, 27 (31.8%) of whom experienced AKI following surgery. Compared to patients without DM or ACEI use, the risk of stage II AKI was nearly three-fold greater among patients with DM who used ACEIs (P < 0.05). The number of stage II AKI patients was very small for multivariable analysis.

**Table 1.** Characteristics of 627 Who Patients Underwent Total Knee Replacement According to Incidence of Acute Kidney Injury^a^

Characteristic	AKI (n = 137), No. (%)	No AKI (n = 490), No. (%)	P Value
**Age, mean ± SD**	71.1 ± 10.2	66.1 ± 10.2	< 0.001
**Sex**			NS
Female	85 (20.3)	334 (79.7)	
Male	52 (25.0)	156 (75.0)	
**Year**			0.002
2008	79 (27.3)	210 (72.7)	
2009	58 (17.2)	280 (82.8)	
**DM**			0.031
Yes	43 (28.1)	110 (71.9)	
No	94 (19.8)	380 (80.2)	
**ACEI/ARB use** ^**b**^			0.028
Any	55 (27.0)	149 (73.0)	
None	81 (19.2)	340 (80.0)	

^a^ Abbreviations: AKI, acute kidney injury; NS, nonsignificant; DM, diabetes mellitus; ACEI, angiotensin converting enzyme inhibitors; ARB, angiotensin receptor blocker.

^b^ Patients with unknown ACEI exposure (one with AKI, one without AKI) were excluded.

**Table 2.** Frequency of Acute Kidney Injury Stages Among the Participants and Mutually Exclusive Categories of Diabetes and angiotensin converting enzyme inhibitors Exposure^a,b^

Exposure	AKI		No AKI
	Stage I	Stage II		
**Total**	120 (19.1)	17 (2.7)	490 (78.1)
**None**	59 (16.7)	7 (2.0)	288 (81.4)
**DM Alone**	13 (19.4)	2 (3.0)	52 (77.6)
**ACEI Use Alone**	25 (21.0)	3 (2.5)	91 (76.5)
**DM + ACEI Use**	22 (25.9)	5 (5.9)	58 (68.2)
**Unknown ACEI Exposure **	1 (50)	0 (-)	1 (50)

^a^ Abbreviations: AKI, acute kidney injury; DM, diabetes mellitus; ACEI, angiotensin converting enzyme inhibitors.

^b^ Data are presented as No. (%).

## 5. Discussion

With the recent trend towards minimally invasive total joint arthroplasty and the increased emphasis on faster recovery and shorter hospital stays, it has become increasingly important to recognize the timing and severity of the various complications associated with elective total joint arthroplasty. Complications following TKR usually occur within the time frame of the typical hospital stay. AKI is a complication that occurs frequently in hospitalized patients. The incidence of AKI in the hospitalized elderly has increased over the past decade and is associated with a substantial increase in morbidity and mortality (25-27). Renal impairment is significantly associated with lengthened duration of hospital stay and one-year all-cause mortality (22, 28, 29). Long-term mortality is greater in patients who survived AKI in comparison to critically or non-critically ill patients without AKI (30, 31). Outcomes are related directly to the severity of AKI, whether characterized by nominal or percentage changes in serum Cr. The higher the duration and stage of AKI are, the higher the long-term morbidity and mortality rates will be (32).

Age, high-risk surgery, DM, metabolic syndrome, use of radiographic contrasts, peripheral vascular disease, alternative medicine drugs, perioperative blood transfusions, general anesthesia, HIV infection, ischemic heart disease, diuretic use, and congestive heart disease are considered risk factors for the development of AKI (16, 33-44). In all the multivariate studies evaluating the risk factors for AKI, age has been consistently seen as potential risk factor (41). In our study, there was a wide variation in the extent of AKI depending on the age. Patients older than 85 years of age were more likely to have severe AKI. In contrast, patients older than 65 years were more likely to develop mild AKI. Obesity and oxidative stress are also independent predictors of AKI (30, 45). Moreover, some ethnic groups are more predisposed to AKI than others (46). This retrospective study showed a statistically significant association between preoperative comorbidities and postoperative AKI in elective TKR surgeries. The incidence of AKI is variable depending on whether the patient has DM or is currently on ACEI or both (34). There is a direct association with AKI if the patient has DM and is on ACEI. DM or ACEI by themselves were not directly implicated in the pathogenesis of AKI.

In our study, the AKI was relatively mild with detecting only AKI stage I and II and no stage III in patients. The perioperative risk stratification could be helpful in anticipating at risk population who could develop AKI after major surgeries (16). Adoption of perioperative renal protection strategies can be extremely helpful in all the patients, including the high-risk ones, undergoing TKR and other joint replacement surgeries. Diagnosis of AKI in the hospitalized elderly can be tricky. All the criteria defining AKI are based on Cr; however, in elderly with low muscle mass, there may be surprisingly slow increase in serum Cr. Novel biomarkers of the tubulointerstitial injury akin to the ones used in myocardial infarction would make the diagnosis of AKI easier in the elderly (41, 47).

This study had some limitations. The factors other than DM, age, and ACEI use, which could possibly precipitate acute renal failure, could not be controlled; however, we believe that factors such as intraoperative hypotension might be a consequence of ACEI use in patients with DM rather than a simple confounder. In addition, we used AKIN to diagnose and quantify AKI in the postoperative settings. AKIN may lead to overdiagnosis of AKI in the elderly where a rise in serum Cr is not corrected for fluid balance in the postoperative settings (48). Therapeutic intervention and renal replacement therapy have minimal impact on the AKI. Resources should focus on renal risk stratification and preventing AKI in the elderly in the perioperative settings (41, 49, 50). Clinicians should be able to anticipate AKI on the basis of patients’ clinical characteristics and minimize the morbidities occurring with postoperative renal failure (51). Strategies should also include modifications in the techniques of anesthesia and titration in addition to practicing renal protection techniques during perioperative period (2, 52-54).

Onuigbo et al. stated that for renal protection in the elderly with underlying CKD, it is reasonable to discontinue ACEI/ARB two to four days prior to elective surgeries or invasive procedures with risk of AKI (55). The same seems to be correct in the elderly who are undergoing elective joint surgeries and are at risk for AKI. In addition, preoperative screening should assess the underlying CKD and risk factors that predisposes to perioperative AKI. Euvolemia should be maintained and all nephrotoxic medications should be preferably avoided in this population (2, 56, 57). Use of preoperative statins as a means to reduce AKI is still under examination and needs further assessment to make any firm recommendations (58). Multidisciplinary approach in which the tandem of the surgeon, anesthesiologist, and internist approach the patient to minimize the perioperative AKI would be reasonable.

In conclusion, we report a retrospective cohort study which reinforces the need for perioperative renal risk stratification especially in the elderly population with DM. This would be cost-effective and would improve morbidity and mortality associated with AKI in the elderly. Further prospective, randomized, double blind studies are needed to substantiate these findings further.
